# Spectral Domain Optical Coherence Tomography in Diffuse Unilateral Subacute Neuroretinitis 

**DOI:** 10.1155/2011/285296

**Published:** 2011-08-11

**Authors:** Carlos Alexandre de A. Garcia Filho, Ana Claudia Medeiros de A. G. Soares, Fernando Marcondes Penha, Carlos Alexandre de Amorim Garcia

**Affiliations:** ^1^Departamento de Oftalmología, Universidade Federal do Rio Grande do Norte, 59072-970 Natal, RN, Brazil; ^2^Departamento de Oftalmología, Universidade Federal de São Paulo, 04021-001 São Paulo, SP, Brazil; ^3^Departamento de Oftalmología, Santa Casa de Misericordia de São Paulo, 01221-020 São Paulo, SP, Brazil

## Abstract

*Purpose*. To describe the SD-OCT findings in patients with diffuse unilateral subacute neuroretinitis (DUSN) and evaluate CRT and RNFL thickness. *Methods*. Patients with clinical diagnosis of DUSN who were submitted to SD-OCT were included in the study. Complete ophthalmologic examination and SD-OCT were performed. Cirrus scan strategy protocols used were 200 × 200 macular cube, optic nerve head cube, and HD-5 line raster. *Results*. Eight patients with DUSN were included. Mean RNFL thickness was 80.25 *μ*m and 104.75 *μ*m for affected and normal eyes, respectively. Late stage had mean RNFL thickness of 74.83 *μ*m compared to 96.5 *μ*m in early stage. Mean CMT was 205.5 *μ*m for affected eyes and 255.13 *μ*m for normal fellow eyes. *Conclusion*. RNFL and CMT were thinner in DUSN eyes compared to normal eyes. Late-stage disease had more pronounced thinning compared to early-stage patients. This thinning in RNFL and CMT may reflect the low visual acuity in patients with DUSN.

## 1. Introduction

Diffuse unilateral subacute neuroretinitis (DUSN) is an inflammatory and infectious disease characterized by insidious and usually severe loss of peripheral and central vision [1]. Clinical features are manifested in early and late stages [1]. The acute phase is characterized by swelling of the optic disc, vitritis, and recurrent crops of evanescent, multifocal, white-yellowish lesions at the outer retina and choroid [2]. The chronic phase presents with optic nerve atrophy, narrowing of retinal vessels and focal or diffuse pigmentary changes [3]. Parasites of different sizes and several species of nematodes have been reported as the etiology of DUSN, including *Toxocara canis, Baylisascaris procyonis, *and* Ancylostoma caninum*, but most of these reports do not present conclusive evidence [4, 5].

Optical coherence tomography (OCT) is a noncontact, noninvasive diagnostic technique that allows measurement of central retinal thickness (CRT) retinal nerve fiber layer (RNFL) thickness and provides important information about the anatomy of the retina and choroid. The development of spectral domain OCT (SD OCT) considerably improved retinal imaging.

The purpose of this study is to describe SD-OCT findings in patients with DUSN and evaluate CRT and RNFL thickness with this image device.

## 2. Patients and Methods

This is a retrospective study in which a medical record review was performed at the Department of Ophthalmology, Federal University of Rio Grande do Norte, Brazil between January 2010 and January 2011. The study was approved by the institutional Review Board, and informed consent was obtained from all patients. Subjects with a diagnosis of DUSN were identified. Eyes included in the study had a minimum of 3 months followup. Patients had clinical diagnosis of DUSN based on Gass and Scelfo criteria, and both early-stage and late-disease patients who underwent SD-OCT (Carl Zeiss Meditec, Dublin, Calif) were included. Any other ocular disease was considered exclusion criteria.

All patients were submitted to complete ophthalmologic examination, including best corrected visual acuity (BCVA), slit lamp examination, tonometry, fundoscopy, and optical coherence tomography with cirrus. Strategy protocols used to obtain images were macular cube 200 × 200 for the central retinal thickness map, optic nerve head cube for retinal nerve fiber layer (RNFL) analysis, and HD-5 line raster to observe macular and foveal aspects.

The following data were collected and recorded: age, sex, initial and final best correct visual acuity (BCVA), affected eye, disease stage, and presence of the worm. Statistical analysis was performed using *Paired Student's t-test*.

## 3. Results

A total of 8 patients with clinical diagnosis of DUSN were included in the study. Mean age of affected patients was 17 years (13–25 yr). Out of 8 patients, 7 were male. Late-stage disease was found in 6 patients. The subretinal worm was identified in only 3 patients, 2 were in early-stage disease and 1 in the chronic stage. All patients in whom the worm was identified were treated with photocoagulation to destroy it.

Table 1 summarizes clinical and OCT findings for all patients included in the study.

Mean RNFL thickness of the affected eyes was 80.25 *μ*m compared to 104.75 *μ*m in the normal contralateral eyes (*P* = 0.0005). There was a difference in RNFL thickness measurements when the early-stage disease (mean RNFL thickness = 96.5 *μ*m) was compared to late-stage disease (mean RNFL thickness = 74.83 *μ*m). 

Central macular thickness was assessed using macular cube strategy. Mean central macular thickness of affected eyes was 205.5 *μ*m compared to 255.13 *μ*m in the normal contralateral eyes (*P* = 0.0004). Macular thickness was similar in early- and late-stage disease (207.5 *μ*m and 204.8 *μ*m, resp.).

With respect to foveal anatomy, 6 out of 8 patients had alterations in foveal contour with a loss of foveal depression, despite diminished central retinal thickness. Neither the early- or late-stage disease patients presented focal or diffuse defects at the junction of the inner and outer segments of photoreceptors (IS/OS junction). 

### 3.1. Cases Report

**Figure 1:**
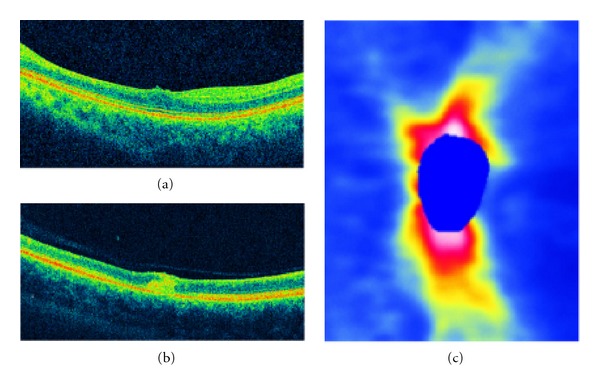
(a) Abnormal foveal architecture in a patient with early-stage DUSN. (b) B-scan in the area the worm was located showing an increased intraretinal reflectivity corresponding to the worm and the surrounded inflammatory reaction. (c) RNFL map with a diffuse thinning.

Case  1 is a 14-year-old male patient in early-stage disease with a 15-day history of low visual acuity in his left eye. Visual acuity was 20/20 in the right eye and counting fingers in the left eye. Biomicroscopy revealed mild vitritis. Fundus examination revealed multifocal, evanescent, white-yellowish lesions near the superior temporal arcade and the presence of a subretinal worm adjacent to the lesions. The worm was destroyed using photocoagulation and visual acuity improved to 20/60.  Figure 1 illustrates SD-OCT findings for this patient.  Figure 1(a) shows abnormal foveal architecture with a thinning in central macular thickness (201 *μ* compared to 246 *μ* in the fellow eye).  Figure 1(b) shows increased intraretinal hyperreflectivity in the area in which the subretinal worm was located. Figure 1(c) shows the RNFL map with a diffuse thinning. Average thickness is 87 *μ* compared to 105 *μ* in the normal fellow eye.

**Figure 2:**
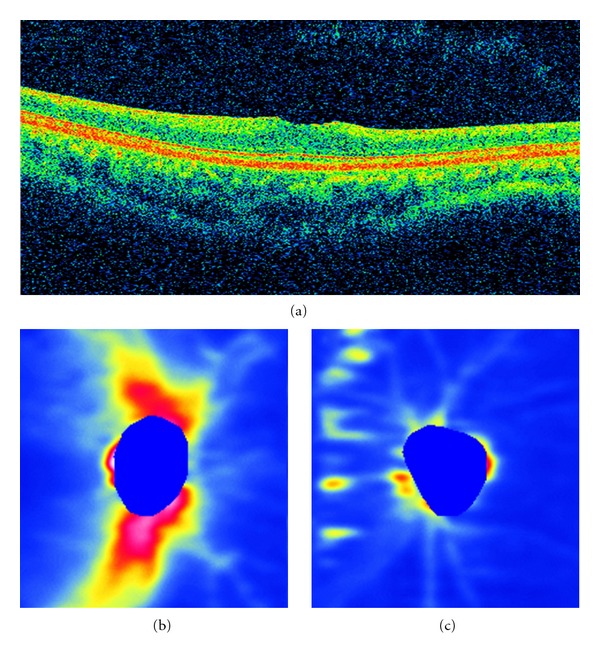
(a) B-scan in the foveal area in a patient with late-stage DUSN presenting a reduced macular thickness and loss of normal foveal contour. RNFL thickness map in the normal eye (b) and a diffuse and pronounced RNFL thinning in the affected eye (c).

Case  7 is a 23-year-old male patient in late-stage DUSN who presented with a 6-month history of low visual acuity. Initial visual acuity was 20/20 in the right eye and counting finger in the left. Fundus examination revealed optic nerve pallor, narrowing of vessels, and diffuse pigmentary changes. The subretinal worm was identified in the nasal retina, and prompt laser photocoagulation was performed. Despite treatment, visual acuity remained counting fingers 90 days after treatment. SD-OCT features for this patient are presented in Figure 2. Figure 2(a) shows the B-scan in the foveal area, with reduced macular thickness and loss of normal foveal contour. Figures 2(b) and 2(c) illustrates the RNFL map in the normal eye and a diffuse and pronounced RNFL thinning in the affected eye.

## 4. Discussion

DUSN is an infectious disease caused by a subretinal nematode leading to inflammation and degeneration of the retina and retinal pigment epithelium. The pathogenesis of DUSN appears to involve a local toxic tissue effect on the outer retina caused by products released by the worm and a diffuse toxic reaction involving inner and outer retinal layers [1]. 

This toxic reaction resulting in inflammation may lead to retinal, RNFL, and optic nerve damage. Previous studies reported a reduction in RNFL thickness in patients with late-stage DUSN using the GDx nerve fiber analyzer [6] and Stratus OCT [7, 8]. In a study with 38 patients diagnosed with late-stage DUSN, Gomes et al. reported a significant decrease in RNFL thickness and a correlation with the low visual acuity presented by these patients [7]. Casella et al. reported the presence of RNFL atrophy even in patients with good visual acuity [8]. Cunha et al. reported an intraretinal worm using a Stratus OCT [9].

The ability of SD-OCT to acquire high-speed (at least 20,000 A-scans per second, compared to 400 A-scans per second for the tome domain OCT), high-resolution (axial resolution of 5 *μ*, compared to 10 *μ* in the Stratus OCT), and high-density three-dimensional images of the macula allows the capture of real retinal geometry that is less affected by eye movements. The high-density, averaged B-scans can be used to evaluate subtle changes in the retinal anatomy [10]. 

In our study, we assessed RNFL thickness in both early- and late-stage DUSN with SD-OCT. All patients in late stage disease presented with a significant decrease in RNFL thickness, and this correlates with the low visual acuity found in these patients (Table 1). All patients in early stage improved visual acuity after treatment. Case  1 improved from counting fingers to 20/60, while patient 2 achieved final visual acuity of 20/25. RNFL was reduced in patient 1 and normal in patient 2. SD-OCT retinal nerve fiber layer map also correlates with retinal thinning (Figures 2(b) and 2(c)). There seems to be a difference between RNFL thickness values between early- and late-stage disease, but as the number of patients in early-stage disease was small, it was not possible to perform statistical analysis. 

Central macular thickness was also assessed in this case series. All patients, including early- and late-stage disease, presented with thinning in the central macular area measurement compared to the normal fellow eye. Early- and late-stage disease had similar values in CMT. Foveal appearance was abnormal in 6 patients. There was thinning in CMT, and the foveal depression was absent.

The worm was identified in only 3 patients (2 in early stage and 1 in late stage), but we were able to perform scans over the area in which the worm was located in only 1 patient. The precise location of the worm could not be identified, but intraretinal hyperreflectivity can be seen in Figure 1(b). This may correspond to the worm and surrounded inflammation caused by its presence.

 Despite the small number of cases, the study showed clear changes in RNFL and macular thickness caused by the releases of worm toxins.

Although SD-OCT may help in identifying RNFL and CMT thinning in patients with DUSN and that these findings correlate with disease stage and visual acuity in these patients, the diagnosis of this condition is still based on the clinical features.

## Figures and Tables

**Table 1:** Clinical data of patients with DUSN and SD-OCT findings.

	Stage of disease	Age	Gender	Eye	RNFL thickness	RNFL fellow eye	CMT	CMT fellow eye	Foveal aspect	Initial VA	Final VA	Worm
Case 1	Early	14	Male	OS	87 *μ*	105 *μ*	201 *μ*	246 *μ*	No foveal depression	CF	20/60	Present
Case 2	Early	15	Male	OD	106 *μ*	112 *μ*	214 *μ*	249 *μ*	Normal foveal depression	20/200	20/25	Present
Case 3	Late	10	Female	OS	88 *μ*	113 *μ*	184 *μ*	259 *μ*	No foveal depression	CF	CF	Absent
Case 4	Late	25	Male	OD	67 *μ*	92 *μ*	217 *μ*	247 *μ*	No foveal depression	CF	CF	Absent
Case 5	Late	19	Male	OD	82 *μ*	102 *μ*	228 *μ*	253 *μ*	No foveal depression	20/200	20/60	Absent
Case 6	Late	17	Male	OS	87 *μ*	114 *μ*	256 *μ*	285 *μ*	Normal foveal depression	HM	HM	Absent
Case 7	Late	23	Male	OS	49 *μ*	100 *μ*	163 *μ*	253 *μ*	No foveal depression	CF	CF	Present
Case 8	Late	13	Male	OD	76 *μ*	100 *μ*	181 *μ*	249 *μ*	No foveal depression	CF	CF	Absent
Mean	NA	17	NA	NA	80.250 *μ*	104.75 *μ*	205.5 *μ*	255.13 *μ*	NA	NA	NA	NA

RNFL: retinal nerve fiber layer.

CMT: central macular thickness.

CF: counting fingers.

HM: hand motion.

NA: not applicable.
